# Income and education predict elevated depressive symptoms in the general population: results from the Gutenberg health study

**DOI:** 10.1186/s12889-019-6730-4

**Published:** 2019-04-24

**Authors:** Jasmin Schlax, Claus Jünger, Manfred E. Beutel, Thomas Münzel, Norbert Pfeiffer, Philipp Wild, Maria Blettner, Jasmin Ghaemi Kerahrodi, Jörg Wiltink, Matthias Michal

**Affiliations:** 1 Department of Psychosomatic Medicine and Psychotherapy, University Medical Center of the Johannes Gutenberg-University Mainz, Untere Zahlbacher Str. 8, 55131 Mainz, Germany; 2 Institute of Medical Biostatistics, Epidemiology & Informatics, University Medical Center of the Johannes Gutenberg-University Mainz, Mainz, Germany; 3 Department of Cardiology I, University Medical Center of the Johannes Gutenberg-University Mainz, Mainz, Germany; 4 Preventive Cardiology and Preventive Medicine, Department of Medicine II, University Medical Center of the Johannes Gutenberg-University Mainz, Mainz, Germany; 5 Department of Ophthalmology, University Medical Center of the Johannes Gutenberg-University Mainz, Mainz, Germany; 6 Center for Thrombosis and Hemostasis, University Medical Center of the Johannes Gutenberg University Mainz, Mainz, Germany; 7 German Center for Cardiovascular Research (DZHK), partner site Rhine-Main, University Medical Center of the Johannes Gutenberg-University Mainz, Mainz, Germany

**Keywords:** Depression, Socioeconomic status, SES, Education, Occupation, Income

## Abstract

**Background:**

Socioeconomic status (SES) has a strong association with depression or physical and mental health in general. However, as SES is a multifaceted construct these associations are not easy to explain. Further, there are several indicators and many studies only investigating two or less indicators at the same time. Therefore, this study aims to analyze the cross-sectional and longitudinal association of three defined SES dimensions (education, occupational position and household net-income) with the occurrence of elevated symptoms of depression relative to the impact of important covariates.

**Methods:**

The study included observational data from 12,484 participants of the Gutenberg Health Study. The outcome was “elevated depressive symptoms” as defined by Patient Health Questionnaire (PHQ-2) ≥ 2 at the 2.5-year follow-up. Regression coefficients were adjusted for baseline covariates (age, sex, partnership, depression, anxiety, medical history of depressive or anxiety disorder and major medical diseases (MMD)) in addition to SES sum score and the three single indicators. We further examined interaction terms of the SES with sex, partnership and major medical diseases. We analyzed the sample stratified by elevated depressive symptoms at baseline, as we expected different trajectories in both subgroups.

**Results:**

SES, education and household net-income were lower in the group of persons with PHQ-2 ≥ 2 at baseline, and they predicted the occurrence of PHQ-2 ≥ 2 at 2.5 year follow-up in the group of persons without elevated depressive symptoms at baseline after multivariable adjustment (SES: Odds Ratio (OR) 0.96, 0.95–0.98, *p* <  0.0001; education: OR 0.96, 0.93–0.99, *p* = 0.036; household net-income: OR 0.96, 0.92–0.99, *p* = 0.046) but not in the group of persons with elevated depressive symptoms at baseline. Further, we found that the impact of major medical diseases on the development of elevated depressive symptoms was buffered by high income. In addition, living in a partnership buffered the impact of a low occupational position.

**Conclusions:**

Regarding the SES, the dimensions education and household net-income seem to play the most important role for socioeconomic inequalities in persons in Mid-West Germany with depressive symptoms.

**Trial registration:**

Reference no. 837.020.07; original vote: 22.3.2007, latest update: 20.10.2015

**Electronic supplementary material:**

The online version of this article (10.1186/s12889-019-6730-4) contains supplementary material, which is available to authorized users.

## Background

Socioeconomic status (SES) is a combined measure of a person’s social position regarding education, occupation and income. There is strong evidence for socioeconomic inequalities in physical and mental health [1]: Persons with lower SES are more likely to develop medical diseases (e.g. diabetes or coronary heart disease) and mental disorders (e.g. depression), are less likely to have access to specialized care and more prone to unfavourable outcome [2–5]. These associations of SES and health have been demonstrated in different subgroups regarding age or culture. Different mediating factors such as psychosocial stress, negative emotions or adverse life events are supposed to contribute to socioeconomic health inequalities [6–11]. Despite the vast amount of studies, the explanation of these associations is limited due to the heterogeneity of SES measures. Further, combined measures obscure the role of the single SES dimensions (e.g. education versus income versus occupation).

Major depression and subthreshold depressive symptoms impair quality of life, increase use of health services and expenditures, and reduce life expectancy [12, 13]. Therefore, studies are needed, which identify subgroups with an increased risk for depressive symptoms. Regarding the importance of SES for depression, Lorant and colleagues [4] summarized the results of 56 studies comprising *n* = 215,490 participants investigating single dimensions of SES and found a higher incidence and persistence of depressive symptoms in persons with lower scores of SES, especially for the dimensions education and income [4]. However, the studies included only investigated one dimension at a time, thus no conclusions about the importance of specific SES factors could be drawn. A Canadian study analyzed the influence of education and household income (amongst others) on major depressive episodes. They found that low education level was associated with a higher risk of major depressive episodes, but only in employed participants. In those who had not worked during the previous 12 months, low education was a “protective” factor which shows the importance of searching for interactions with explanatory worth. In both groups income was negatively associated with the risk of major depressive episodes [14]. However, this study included neither occupational position nor baseline depression as covariates. Similar results with assimilable limitations have been found in the Longitudinal Aging Study Amsterdam [15]. One major limitation of previous studies on the relationship of SES with depression is that they – to the best of our knowledge – did not include baseline depression as covariates or included all three recommended SES dimensions [16].

Hence, we want to determine a) the associations between the main SES factors and elevated depressive symptoms at baseline and after 2.5 years in a population-based sample in Mid-West-Germany and b) whether the contribution of SES is independent from common predictors of depression (e.g. baseline depression, medical history of depression or anxiety, sociodemographic variables, major medical diseases (MMD)) and c) whether there are any interactions of the SES factors with sex, partnership or MMD in persons from this area. The results will extend the knowledge about socioeconomic inequalities in depression.

## Methods

### Study sample

This study analyzed data from the first 15,010 participants in the Gutenberg Health study (GHS). The GHS is a population-based, prospective, observational single-center in western Mid-Germany with an age range of 35 to 74 years. Exclusion criteria were insufficient ability in German language and physical and mental disability to participate. The sample had been stratified for sex, residence and decades of age. The study protocol and study documents were approved by the local ethics committee of the Medical Chamber of Rhineland-Palatinate, Germany (reference no. 837.020.07; original vote: 22.3.2007, latest update: 20.10.2015) and by the local and federal data safety commissioners.

In the present study longitudinal data from baseline assessment and 2.5 year follow-up have been used. From *n* = 15,010 participants, *n* = 315 were excluded due to missing baseline data of depression, *n* = 1360 due to missing baseline data of SES (or SES factors), and *n* = 1165 due to missing follow-up data of depression thus leaving finally *n* = 12,484 participants to be analyzed. For the characteristics of the sample see Table 1.

**Table 1:** Characteristics of the sample stratified by being bothered by symptoms of depression (PHQ-2 ≥ 2)

	Total sample (*n* = 12,484)	Comparison		p
		PHQ-2 < 2 (9605/12484)	PHQ-2 ≥ 2 (2879/12484)	
Age, years, mean ± SD	54.6 ± 10.9 (12484)	54.9 ± 11.0 (9605)	53.4 ± 10.6 (2879)	< 0.0001*
Female, % (N)	48.5% (6054/12484)	46.4% (4456/9605)	55.5% (1598/2879)	**< 0.0001**
Living with partner, yes, % (N)	82.1% (10,249/12483)	84% (8067/9604)	75.8% (2182/2879)	**< 0.0001**
*School education, % (N)*				
9 years	37.4% (4665/12484)	37.1% (3564/9605)	38.2% (1101/2879)	0.139
10 years	23.4% (2919/12484)	23.2% (2230/9605)	23.9% (689/2879)	0.221
12–13 years	38.5% (4806/12484)	39% (3743/9605)	36.9% (1063/2879)	**0.025**
other school graduations	0.4% (55/12484)	0.4% (40/9605)	0.5% (15/2879)	0.280
no graduation	0.3% (39/12484)	0.3% (28/9605)	0.4% (11/2879)	0.276
*Professional education, % (N)*				
apprenticeship/vocational school	46.1% (5759/12484)	45.3% (4351/9605)	48.9% (1408/2879)	**< 0.0001**
master/technical school	15.4% (1926/12484)	15.6% (1499/9605)	14.8% (427/2879)	0.163
university/university of applied sciences	29.9% (3730/12484)	31% (2981/9605)	26% (749/2879)	**< 0.0001**
other professional graduations	2.7% (336/12484)	2.7% (258/9605)	2.7% (78/2879)	0.499
without professional graduation	5.9% (733/12484)	5.4% (516/9605)	7.5% (217/2879)	**< 0.0001**
*Employment status, % (N)*				
no	37.1% (4635/12482)	37.7% (3622/9603)	35.2% (1013/2879)	**0.007**
yes, full-time employed	45.8% (5720/12482)	46% (4413/9603)	45.4% (1307/2879)	0.307
yes, part-time employed	13.4% (1670/12482)	13% (1245/9603)	14.8% (425/2879)	**0.007**
yes, marginally employed	3.7% (457/12482)	3.4% (323/9603)	4.7% (134/2879)	**0.001**
SES^1^ (3–21), mean ± SD	13.2 ± 4.4 (12484)	13.4 ± 4.4 (9605)	12.5 ± 4.2 (2879)	< 0.0001*
Education sum score (1–7), mean ± SD	3.9 ± 2.2 (12484)	3.9 ± 2.2 (9605)	3.8 ± 2.1 (2879)	< 0.0001*
Occupation sum score (1–7), mean ± SD	4.8 ± 1.6 (12484)	4.9 ± 1.6 (9605)	4.6 ± 1.5 (2879)	**< 0.0001**
Household net-income (1–7), mean ± SD	4.5 ± 1.7 (12484)	4.6 ± 1.7 (9605)	4.1 ± 1.8 (2879)	**< 0.0001**
PHQ-2 ≥ 2 at T0, % (N)	23.1% (2879/12484)	–	23.1% (2879/12484)	**–**
PHQ-2 ≥ 2 at T1, % (N)	23.2% (2898/12484)	15.1% (1455/9605)	50.1% (1443/2879)	**< 0.0001**
PHQ-2 score at T0, mean ± SD	0.9 ± 1.0 (12484)	0.4 ± 0.5 (9605)	1.7 ± 1.6 (2879)	< 0.0001*
PHQ-2 score at T1, mean ± SD	0.8 ± 1.2 (12484)	0.6 ± 0.9 (9605)	1.7 ± 1.6 (2879)	< 0.0001*
MH^2^ of depressive disorder, yes, % (N)	11.5% (1433/12472)	7.1% (683/9599)	26.1% (750/2873)	**< 0.0001**
GAD-2 score, mean ± SD	0.9 ± 1.1 (12436)	0.6 ± 0.8 (9569)	1.8 ± 1.4 (2867)	< 0.0001*
MH^2^ of anxiety disorder, yes, % (N)	7.1% (880/12475)	4.7% (447/9601)	15% (433/2874)	**< 0.0001**
CVD^3^, % (N)	6.3% (776/12313)	6.2 (589/9478)	6.6% (187/2835)	0.455
Cancer, % (N)	8.7% (1082/12476)	8.5% (817/9599)	9.2% (265/2877)	0.242
Diabetes, % (N)	8.5% (1062/12484)	8.4% (805/9605)	8.9% (257/2622)	0.361
COPD^4^, % (N)	4.7% (583/12484)	4.2% (408/9605)	6.1% (175/2879)	**< 0.0001**
Asthma, % (N)	2.7% (334/12484)	2.5% (240/9605)	3.3% (94/2879)	**0.028**
Major medical diseases^5^, % (N)	23.5% (2907/12363	22.8% (2186/9513)	25% (721/2850)	**0.011**

Data are described as mean ± standard deviation (n) or percentage with proportional numbers in brackets (n/n)

^1^*SES* Socioeconomic status

^2^*MH* Medical history

^3^*CVD* Cardiovascular diseases, e.g. coronary heart diseases, stroke or medicated heart failure

^4^*COPD* Chronic obstructive lung disease

^5^Major medical diseases = any of the following diseases: *CVD* Cancer, diabetes, *COPD* Asthma

*boldface indicates *p* < 0.5 for odds ratios

### Assessment

After inclusion, participants had been invited to the study center to run through an examination of 5 h duration which aimed an evaluation of classical cardiovascular risk factors and clinical variables. Questionnaires, computer-assisted personal interviews, laboratory and further medical examinations were used. The follow-up assessment was conducted through computer-assisted interviews.

#### Outcome variable

The outcome was “elevated depressive symptoms” after 2.5 years as measured by the 2 item version of the Patient Health Questionnaire depression module (PHQ-2) [17]. The PHQ-2 asks for the frequency of anhedonia (“Little interest or pleasure in doing things”) and depressed mood (“Feeling down, depressed or hopeless”) over the past 2 weeks (“Over the last 2 weeks, how often have you been bothered by …”). It can reach values from 0 to 6 [17]. The PHQ-2 shows a high reliability (α = 0.83) and a high correlation with the longer version PHQ-9 (r = 0.87). A cut-off score of 3 or more has a sensitivity of 79% and a specificity of 86% for any depressive disorder. In the present study we used the cut-off of 2 or more to capture subthreshold depressive symptoms in addition to depressive disorders [18].

#### Predictor variable

The main predictor variables were SES and its components at baseline. SES was calculated according the rationale of the health monitoring of the Robert Koch Institute (RKI) [16]. This SES indicator combines scores for the dimensions school and professional education, occupational position and household net-income [16]. The three components of SES were assigned scores between 1 and 7, thus the SES sum score can reach values from 3 to 21 with 21 indicating the highest SES. To simplify the interpretation we also recoded SES as a categorical variable with SES 3–7.7, 7.8–14.1 und 14.2–21 indicating low, medium and high SES. The threshold scores were used from a former study to enable comparability [19]. The SES dimensions were assessed by a computer-assisted interview. The dimension education includes school and professional education: No school or professional qualification or certificate of primary or secondary education or rather no professional qualification or other lower professional qualifications represent the lowest school and professional qualification with 1.0 point. Technical college qualification (“Fachhochschulreife”), University Entrance Qualification (“Abitur”) or other higher graduations and Bachelor/Technical College Diploma, Master/Magister/Diploma/PhD and other higher vocational qualifications indicate the highest education with 7.0 points. Regarding the occupational position, persons in apprenticeship and un- or semiskilled workers fall in the category of 1.0 point; and freelance academics, civil servants in highest service, self-employed in trading/business/etc. represent the highest occupational position with 7.0 points. The overall monthly household net-income ranges from < 1250 € (1.0 point) to ≥5000 € (7.0 points). For the specific calculation basis for the index of the socioeconomic status see Additional file 1.

#### Covariates

The following covariates were included: Baseline depression according to PHQ-2; anxiety (according to GAD-2); medical history of any depressive or of any anxiety disorder (“Have you ever received the definite diagnosis of any depressive disorder/anxiety disorder by a physician?” yes/no); occurrence of a major medical diseases (MMD) combining any of the following diseases: cardiovascular disease (e.g. coronary heart diseases, stroke, medicated heart failure, cancer, diabetes, chronic obstructive lung disease or asthma). Further covariates were age (in years), sex (male/female), living with a partner (yes/no) which were all were assessed by a computer-assisted interview. Anxiety was measured by the two-item Generalized Anxiety Disorder-Scale (GAD-2) [20, 21]. The GAD-2 asks for the frequency of anxiety (“feeling nervous, anxious or on edge”) and worry control (“not being able to stop or control worrying”) over the past 2 weeks (“Over the last 2 weeks, how often have you been bothered by any of the following problems?”). The GAD-2 score can reach values from 0 to 6 with a cut-off of 3 defining clinical relevant anxiety disorders (e.g. generalized anxiety disorder, social phobia or panic disorder) with a sensitivity of 65% and specificity of 88% [22]. The GAD-2 shows an acceptable reliability (α = 0.75) [23]. In the present study we used the GAD-2 as a dimensional variable.

### Statistical analysis

Baseline group differences (PHQ-2 ≥ 2 yes/no) were examined by two-tailed t-tests and χ^2^-tests. The longitudinal associations were analyzed by logistic regression models with PHQ-2 ≥ 2 yes/no as the dependent variable and SES (or its components) as the main predictor variable. There were two basic models of adjustment. Model 1 included the covariates age, sex and partnership. Model 2 additionally included the severity of baseline anxiety (GAD-2 sum score) and baseline depression (PHQ-2 sum score), medical history of any depressive or anxiety disorder, partnership (yes/no), occurrence of major medical disease. Further, the SES global indicator or one of its subdimensions or all three subdimensions were included as the predictor variable. For exploratory analyses, we examined several possible interactions of SES or its components with sex, partnership and occurrence of a major medical disease. The regression models were calculated stratified for the two subsamples of persons with baseline PHQ-2 < 2 versus PHQ-2 ≥ 2 assuming that different pathways might be important in both groups and for the complete sample. All calculations were made with SPSS 23 (Statistical Package for the Social Sciences, Chicago, Illinois, USA) [24].

## Results

### Characteristics of the sample stratified by PHQ-2 ≥ 2

Of 12,484 participants, 23.1% (*n* = 2879) had elevated depressive symptoms at baseline (PHQ-2 *≥* 2). These persons were less likely to live in a partnership. They were more likely to experience depressive or anxiety symptoms after 2.5 years, to have been diagnosed with depressive or anxiety disorder previously and to suffer from chronic obstructive pulmonary disease (COPD) or asthma. Although there were no significant differences between the groups regarding cardiovascular disease (CVD), cancer or diabetes, the group with elevated depressive symptoms had overall a higher prevalence of MMD. With regard to sociodemographic variables participants with a PHQ-2 score of 2 or more were more likely to be female and younger. Furthermore, they had an overall lower SES (see Table 1).

### Longitudinal association of SES with elevated depressive symptoms at the 2.5 year follow-up

#### SES (global score)

SES predicted the occurrence of elevated depressive symptoms 2.5 years later only in the group of persons without elevated depressive symptoms at baseline (and in the total sample respectively). Each point increase in the SES combined score ranging from 3 to 21 reduced the risk of developing elevated depressive symptoms by 4% in fully adjusted model, respectively by 24% if the categorical variable was used (Table 2).

**Table 2:** Prediction of depressive symptoms at the 2.5 year follow-up (T1) by SES^1^

	Complete sample (T0)		persons without elevated depressive symptoms at T0		persons with elevated depressive symptoms at T0	
	PHQ-2 ≥ 2 at T1 (2898/12484)		PHQ-2 ≥ 2 at T1 (1455/9605)		PHQ-2 ≥ 2 at T1 (1443/2879)	
	Adj. OR (95% CI)	p	Adj. OR (95% CI)	p	Adj. OR (95% CI)	p
*SES* ^1^						
Model 1						
SES^1^ (range 3–21)	**0.96 (0.95–0.97)**	**< 0.0001**	**0.96 (0.95–0.98)**	**< 0.0001**	0.99 (0.97–1.01)	0.176
SES^1^ (low-med-high)	**0.77 (0.72–0.83)**	**< 0.0001**	**0.76 (0.69–0.84)**	**< 0.0001**	0.95 (0.84–1.07)	0.391
Model 2						
SES^1^ (range 3–21)	**0.97 (0.96–0.98)**	**< 0.0001**	**0.96 (0.95–0.98)**	**< 0.0001**	0.98 (0.97–1.00)	0.110
SES^1^ (low-med-high)	**0.81 (0.75–0.88)**	**< 0.0001**	**0.76 (0.69–0.84)**	**< 0.0001**	0.93 (0.82–1.06)	0.265

Model 1 adjusted for sex, age, living with partner and major medical diseases

Model 2 adjusted for sex, age, living with partner, major medical diseases, PHQ-2 (T0), MH^2^ of depressive disorder, GAD-2 (T0) and MH^2^ of anxiety disorder

^1^*SES* Socioeconomic status

^2^*MH* Medical history

^3^Major medical diseases = any of the following diseases: *CVD* Cancer, diabetes, *COPD* Asthma

*boldface indicates *p* < 0.5 for odds ratios

#### Education

Only in the group of persons without elevated depressive symptoms at baseline (and in the total sample respectively), education predicted the occurrence of elevated depressive symptoms in the fully adjusted model (adjusted also for occupational position and household income). Each point increment reduced the risk for the occurrence of elevated depressive symptoms at the 2.5 year follow-up by 4% (see Table 3).

**Table 3:** Prediction of depressive symptoms at the 2.5 year follow-up (T1) by the SES indicator education

	Complete sample (T0)		persons without elevated depressive symptoms at T0		persons with elevated depressive symptoms at T0	
	PHQ-2 ≥ 2 at T1 (2898/12484)		PHQ-2 ≥ 2 at T1 (1455/9605)		PHQ-2 ≥ 2 at T1 (1443/2879)	
	Adj. OR (95% CI)	p	Adj. OR (95% CI)	p	Adj. OR (95% CI)	p
*Education*						
Model 1						
*(A) only*						
Education (range 1–7)	**0.95 (0.93–0.97)**	**< 0.0001**	**0.95 (0.92–0.98)**	**< 0.0001**	0.98 (0.94–1.01)	0.230
*(B) with three dimensions*						
Education (range 1–7)	0.99 (0.96–1.01)	0.269	0.98 (0.95–1.02)	0.300	0.98 (0.94–1.03)	0.432
Model 2						
*(A) only*						
Education (range 1–7)	**0.95 (0.93–0.97)**	**< 0.0001**	**0.94 (0.91–0.97)**	**< 0.0001**	0.97 (0.93–1.01)	0.093
*(B) with three dimensions*						
Education (range 1–7)	**0.97 (0.94–0.99)**	**0.015**	**0.96 (0.93–0.99)**	**0.036**	0.97 (0.93–1.02)	0.206

Model 1 adjusted for sex, age, living with partner and major medical diseases^3^

Model 2 adjusted for sex, age, living with partner, major medical diseases^3^, PHQ-2 (T0), MH^2^ of depressive disorder, GAD-2 (T0) and MH^2^ of anxiety disorder

A: included only the respective SES dimension, e.g. education; B: included all three SES dimensions concurrently as predictor variables

^2^*MH* Medical history

^3^Major medical diseases = any of the following diseases: *CVD* Cancer, diabetes, *COPD* Asthma

*boldface indicates *p* < 0.5 for odds ratios

#### Occupational position

The occupational position did not predict the occurrence of elevated depressive symptoms at the 2.5 year follow-up in the fully adjusted model (adjusted also for the other two SES dimensions, Table 4).

**Table 4:** Prediction of depressive symptoms at 2.5 year follow-up (T1) by the SES indicator occupational position

	Complete sample (T0)		persons without elevateddepressive symptoms at T0		persons with elevated depressive symptoms at T0	
	PHQ-2 ≥ 2 at T1 (2898/12484)		PHQ-2 ≥ 2 at T1 (1455/9605)		PHQ-2 ≥ 2 at T1 (1443/2879)	
	Adj. OR (95% CI)	p	Adj. OR (95% CI)	p	Adj. OR (95% CI)	p
*Occupational position*						
Model 1						
*with single indicator*						
Occupational position (range 1–7)	**0.92 (0.89–0.95)**	**< 0.0001**	**0.92 (0.89–0.96)**	**< 0.0001**	0.98 (0.94–1.03)	0.517
*with three indicators*						
Occupational position (range 1–7)	**0.96 (0.93–0.99)**	**0.018**	0.96 (0.92–1.00)	0.075	1.01 (0.95–1.07)	0.860
Model 2						
*with single indicator*						
Occupational position (range 1–7)	**0.94 (0.91–0.97)**	**< 0.0001**	**0.93 (0.89–0.96)**	**< 0.0001**	0.97 (0.93–1.03)	0.310
*with three indicators*						
Occupational position (range 1–7)	0.97 (0.94–1.01)	0.162	0.97 (0.92–1.01)	0.153	1.00 (0.94–1.06)	0.879

Model 1 adjusted for sex, age, living with partner and major medical diseases^3^

Model 2 adjusted for sex, age, living with partner, major medical diseases^3^, PHQ-2 (T0), MH^2^ of depressive disorder, GAD-2 (T0) and MH^2^ of anxiety disorder

A: included only the respective SES dimension, e.g. education; B: included all three SES dimensions concurrently as predictor variables

^2^*MH* Medical history

^3^Major medical diseases = any of the following diseases: *CVD* Cancer, diabetes, *COPD* Asthma

*boldface indicates *p* < 0.5 for odds ratios

#### Household net-income

Household net-income predicted the occurrence of elevated depressive symptoms in the fully adjusted model (also adjusted for the other two SES dimensions; Table 5). Each increment increase of the household net-income reduced the risk for developing elevated depressive symptoms by 4%.

**Table 5:** Prediction of depressive symptoms at 2.5 year follow-up (T1) by the SES indicator household net-income

	Complete sample (T0)		persons without elevated depressive symptoms at T0		persons with elevated depressive symptoms at T0	
	PHQ-2 ≥ 2 at T1 (2898/12484)		PHQ-2 ≥ 2 at T1 (1455/9605)		PHQ-2 ≥ 2 at T1 (1443/2879)	
	Adj. OR (95% CI)	p	Adj. OR (95% CI)	p	Adj. OR (95% CI)	p
*Household net-income*						
Model 1						
*with single indicator*						
Household net-income (range 1–7)	**0.89 (0.87–0.92)**	**< 0.0001**	**0.91 (0.88–0.95)**	**< 0.0001**	0.97 (0.92–1.02)	0.195
*with three indicators*						
Household net-income (range 1–7)	**0.92 (0.89–0.95)**	**< 0.0001**	**0.94 (0.90–0.98)**	**< 0.0001**	0.97 (0.92–1.03)	0.351
Model 2						
*with single indicator*						
Household net-income (range 1–7)	**0.94 (0.91–0.97)**	**< 0.0001**	**0.92 (0.89–0.96)**	**< 0.0001**	0.98 (0.93–1.03)	0.354
*with three indicators*						
Household net-income (range 1–7)	0.97 (0.94–1.00)	0.062	**0.96 (0.92–0.99)**	**0.046**	0.99 (0.94–1.05)	0.782

Model 1 adjusted for sex, age, living with partner and major medical diseases^3^

Model 2 adjusted for sex, age, living with partner, major medical diseases^3^, PHQ-2 (T0), MH^2^ of depressive disorder, GAD-2 (T0) and MH^2^ of anxiety disorder

A: included only the respective SES dimension, e.g. education; B: included all three SES dimensions concurrently as predictor variables

^2^*MH* Medical history

^3^Major medical diseases = any of the following diseases: *CVD* Cancer, diabetes, *COPD* Asthma

*boldface indicates *p* < 0.5 for odds ratios

### Longitudinal association of interaction terms with depressive symptoms at the 2.5 year follow-up

**Fig. 1:**
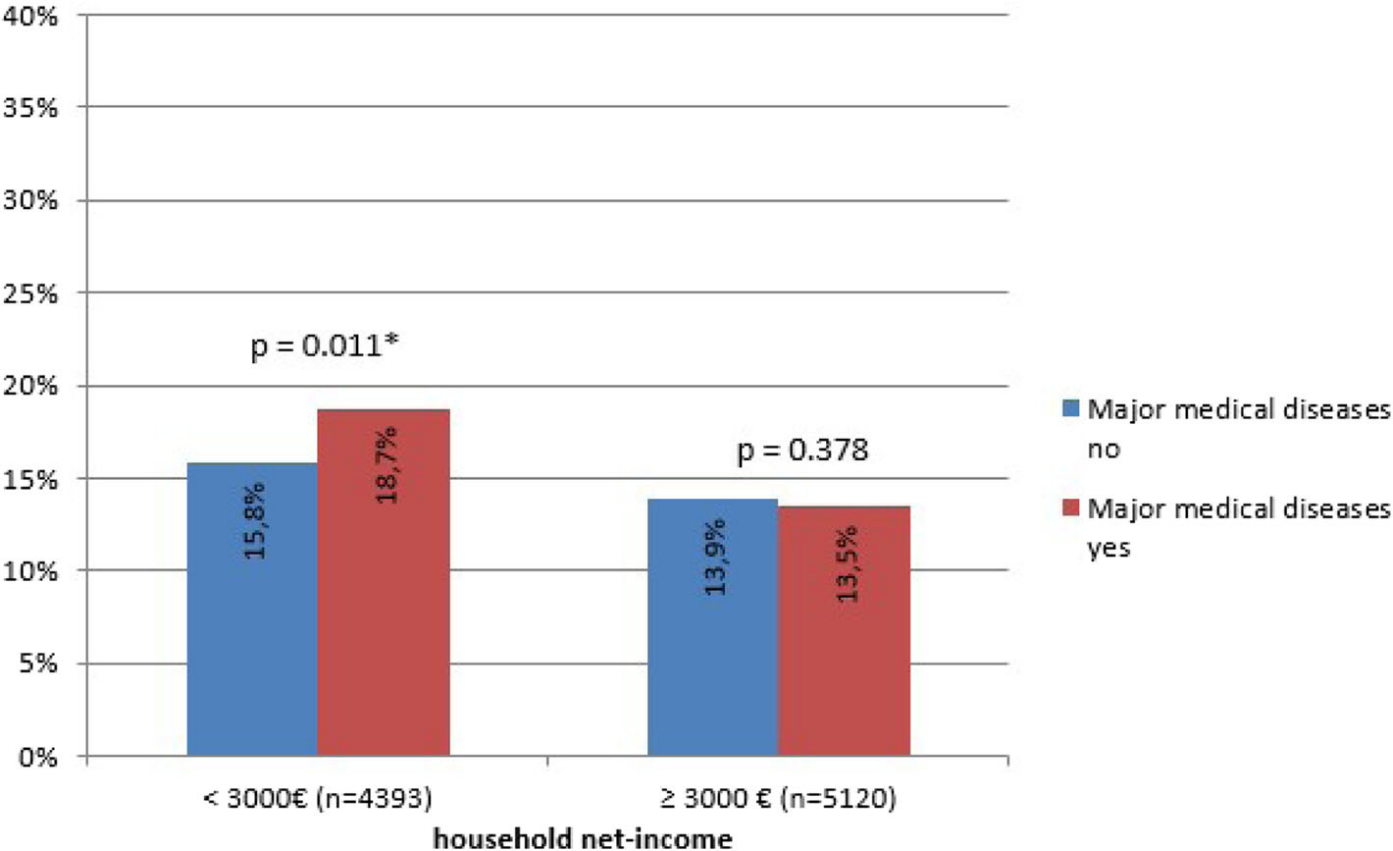
Interaction between household net-income and MMD in the group without depressive symptoms at T0. *Legend.* MMD = major medical disease. PHQ-2 ≥ 2, yes, in %. Differences tested for significance via Pearson chi^2^

In our explorative analysis, we found two interactions (Additional file 2): First, we revealed an interaction between household net-income and major medical disease. In order to simplify the interpretation of the interaction terms, we dichotomized the household net-income (1 to 4 vs. 5 to 7). The interaction between household net-income and major medical diseases showed a trend with marginal significance in the fully adjusted model (Odds Ratio (OR) 0.78, 0.60–1.02): Major medical disease only increased the risk in persons in the low household net-income category (< 3000 €) and not in the group of persons with high income (Fig. 1).

**Fig. 2:**
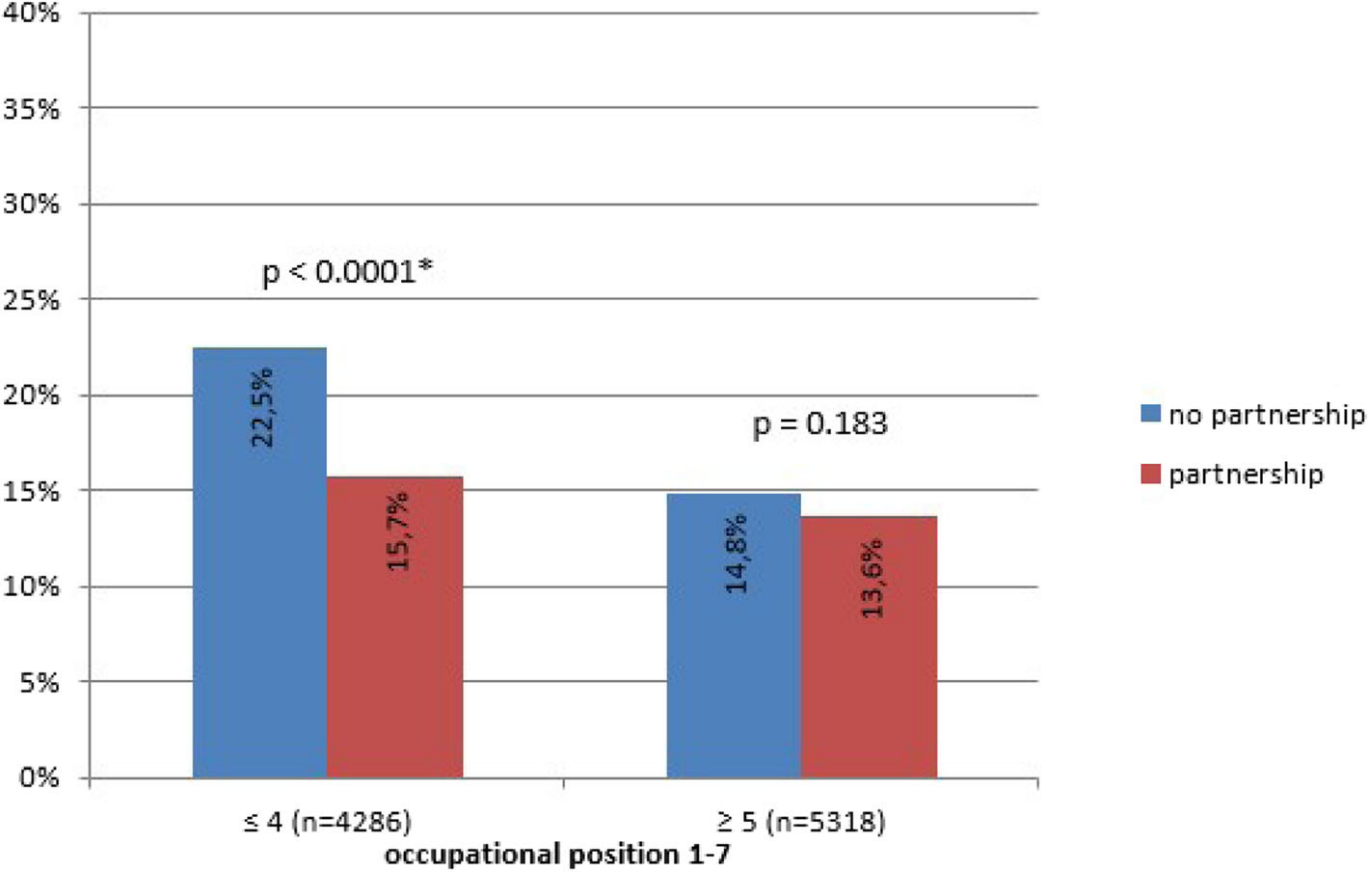
Interaction between occupational position and living with partner in group without depressive symptoms at T0. *Legend.* PHQ-2 ≥ 2, yes, in %. Differences tested for significance via Pearson chi^2^

Second, there was a significant interaction of the occupational position with living in a partnership produced in the full sample (OR 1.30, 1.05–1.62, *p* = 0.018). Living in a partnership buffered the detrimental effect of a lower occupational position on the development of elevated depressive symptoms (Fig. 2).

Further, there was an interaction of household net-income with partnership in the group of persons without depressive symptoms at baseline. However, after full adjustment the interaction disappeared (Additional file 2).

## Discussion

The main findings are: 1) Persons without elevated depressive symptoms at baseline had a higher socioeconomic status (on all three indicators compared to persons with elevated depressive symptoms). 2) 50.1% of the group with elevated depressive symptoms at baseline had elevated depressive symptoms 2.5 years later versus 15.1% of the group without baseline depressive symptoms. 3) In the group without elevated depressive symptoms at baseline, SES and its dimensions income and education were strong and independent predictors of elevated depressive symptoms at the 2.5-year follow-up. In the group of persons with depressive symptoms at baseline, however, SES was not related with the occurrence of elevated depressive symptoms at the 2.5-year follow-up. 5) In the explorative analyses we found interactions of household net-income with major medical diseases as well as occupational position with living with a partner. The detrimental effect of MMD on the development of elevated depressive symptoms was buffered by high income and living in a partnership buffered the detrimental effect of a low occupational position.

Regarding the prediction of elevated depressive symptoms by SES, we found that particularly the dimensions educational qualification and household net-income were important predictors, which is in line with the results of the meta-analysis of Lorant and colleagues [4]. Important to note, the effects of these SES dimensions were as strong as or even stronger than the effects of major medical disease, partnership or sex in our sample (Additional file 3). In contrast to Lorant and colleagues [4], in our sample the influence of SES on the development of elevated depressive symptoms was negligible for persons who were already bothered by depressive symptoms at baseline. This differential effect of SES in the two groups might mean that once a person is depressed, the effect of SES for the further 2.5 year course is negligible in comparison with direct measures of mental health or disease burden. In persons without current elevated depressive symptoms, however, SES represents an important factor. Further reasons for our divergent findings, might be that we applied different baseline measures and included a wide range of relevant covariates (e.g., baseline depression and anxiety, MH of depression or anxiety, MMD, sociodemographic variables vs. only sociodemographic variables, prevalence of depression), that we analyzed the sample stratified by baseline depression, that we used slightly different predictor variables (SES indicators as dimensional variables vs. as binary variables) and that Lorant and colleagues [4] included studies from different countries with different health care systems.

Limitations of our study concern the lack of clinician-administered interviews of depression. However, it has been repeatedly demonstrated that even subthreshold depressive symptoms or elevated depressive symptoms according to the criterion PHQ-2 ≥ 2 impair quality of life, increase use of health services and expenditures, and reduce life expectancy [12, 13, 25], emphasizing the public health relevance of our findings.

Further, the study impresses through its sample size and its representativeness regarding the general population as well as through the standardized and therefore high comparable measurement of the SES indicators as Lampert and Kroll would recommend them [16]. Moreover, we were able to investigate the sample at two times of measurement and include baseline depressive symptoms as group variable and covariate as well as severe medical diseases. In the interpretation of the results, it should be considered that, due to the design, collider stratification may occur with regard to persons with already depressive symptoms at the first measurement point; PMID: 19689488. In this context it can also happen regression to the mean which means that those who start a cohort high are not going to stay high. These issues are due to stratification based on baseline symptoms [26, 27]. Due to the age range of our sample starting with 35 years, conclusions about the direction of the association between depression and SES indicators are limited. We assume that education and occupational position were largely determined at study entrance. Nevertheless, it can be assumed that the SES still varies over time in this age range, which could not be assessed in the present study. However, Gilman and colleagues found that childhood SES is associated with depression in adulthood [28]. Therefore, the results support the argument that causation as a mechanism is more important than selection. It can also be assumed that the effects cannot be applied equally to both sexes. This possible gender effect cannot be given sufficient attention in this paper; it is a research question in itself. In the present study, however, sex has been included as a control variable. It can also be assumed that not all ethnic groups are equally influenced by the SES indicators. This, too, is a research question in its own right, which is investigated in Beutel and colleagues [29], among others. Further, conclusions can be drawn with regard to the population group studied, since it can be assumed that countries differ in terms of the social determinants of health and well-being [30–33].

## Conclusions

In the present study, education and household net-income were independent predictors for the incidence of elevated depressive symptoms in persons from Mid-West-Germany who were free from elevated depressive symptoms at baseline. Occupational position seemed to have no independent longitudinal influence on depressive symptoms in this sample. Therefore, we conclude that education and household net-income might play a causative role for the elevated risk of persons with lower SES to develop depressive symptoms in persons from this area.

## Additional files

Additional file 1: Specific allocations of original SES indicator data to scores with ranges from 1 to 7. (DOCX 26 kb)

Additional file 2: Prediction of depressive symptoms at the 2.5 year follow-up (T1) by the interaction terms. (DOCX 34 kb)

Additional file 3: Prediction of depressive symptoms at the 2.5 year follow-up (T1) by other variables. (DOCX 28 kb)

## Abbreviations

COPD: Chronic obstructive pulmonary disease
CVD: Cardiovascular disease
GAD: Generalized Anxiety Disorder-Scale
MMD: Major medical disease
OR: Odds ratio
PHQ-2: Patient Health Questionnaire, depression module
SES: Socio-economic status
