# Supplementary material for: Income and education predict elevated depressive symptoms in the general population: results from the Gutenberg health study
Source: BMC Public Health. 2019 Apr 24;19:430. doi: 10.1186/s12889-019-6730-4 (PMC6480596; doi:10.1186/s12889-019-6730-4)
Supplement: Supplementary file 1 — Specific allocations of original SES indicator data to scores with ranges from 1 to 7. (DOCX 26 kb) [file 12889_2019_6730_MOESM1_ESM.docx]

**Supplement C** Specific allocations of original SES^1^ indicator data to scores with ranges from 1 to 7.

| **Score** |  |  | **school and professional education** | | | |  | **occupational position** | |  | **household net-income in €** | |
| --- | --- | --- | --- | --- | --- | --- | --- | --- | --- | --- | --- | --- |
|  |  | original data school | | original data profession | | n/N^2^ |  | original data | n/N |  | original data | n/N |
| 1 |  | certificate of primary or secondary education, other or no graduation | | other vocational qualification, no vocational qualification (, Bachelor/Technical College Diploma, Master/Magister/Diploma/PhD) | 612/12484 | |  | un- or semi-skilled workers, in apprenticeship | 141/12484 |  | < 1250 € | 792/12484 |
| 2 |  | certificate of primary education, other or no graduation | | training/apprenticeship/vocational school, master/ technical school, other vocational qualification | 4199/12484 | |  | self-employed farmer, un- or semi-skilled workers | 1194/12484 |  | 1250 € to < 1750 € | 1158/12484 |
| 3 |  | certificate of secondary education, Technical college qualification (“Fach-hochschulreife”), University Entrance Qualification (“Abitur”) | | training/apprenticeship/vocational school, master/ technical school, other vocational qualification, no vocational qualification | 2740/12484 | |  | civil servants/judge/professional soldier, employee, un- or semi-skilled workers | 1089/12484 |  | 1750 € to < 2250 € | 1625/12484 |
| 4 |  | other school graduations | | training/apprenticeship/vocational school, master/ technical school, other vocational qualification | 24/12484 | |  | civil servants/judge/professional soldier, employee, family workers | 3403/12484 |  | 2250 € to < 3000 € | 2494/12484 |
| **Score** |  | **school and professional education** | | | | |  | **occupational position** | |  | **household net-income in €** | |
|  |  | original data school | | original data profession | n/N | |  | original data | n/N |  | original data | n/N |
| 5 |  | certificate of primary or secondary education, Technical college qualification (“Fach-hochschulreife”), University Entrance Qualification (“Abitur”) | | training/apprenticeship/vocational school, master/ technical school, Bachelor/Technical College Diploma, Master/Magister/Diploma/PhD, other vocational qualification | 1396/12484 | |  | self-employed in trading/business/etc. | 907/12484 |  | 3000 € to < 4000 € | 2904/12484 |
| 6 |  | Technical college qualification (“Fach-hochschulreife”), University Entrance Qualification (“Abitur”), other graduations | | other vocational qualification | 2/12484 | |  | freelance academics, civil servants/judge/professional soldier, employee | 4334/12484 |  | 4000 € to < 5000 € | 1504/12484 |
| 7 |  | Technical college qualification (“Fach-hochschulreife”), University Entrance Qualification (“Abitur”), other graduations | | Bachelor/Technical College Diploma, Master/Magister/Diploma/PhD, other vocational qualification | 3511/12484 | |  | freelance academics, self-employed in trading/business/etc., civil servants/judge/professional soldier, employee | 1416/12484 |  | ≥ 5000 € | 2007/12484 |

^1^SES = socioeconomic status.

^2^n/N = sample size partial/whole sample.
