# Supplementary material for: Income and education predict elevated depressive symptoms in the general population: results from the Gutenberg health study
Source: BMC Public Health. 2019 Apr 24;19:430. doi: 10.1186/s12889-019-6730-4 (PMC6480596; doi:10.1186/s12889-019-6730-4)
Supplement: Supplementary file 2 — Prediction of depressive symptoms at the 2.5 year follow-up (T1) by the interaction terms. (DOCX 34 kb) [file 12889_2019_6730_MOESM2_ESM.docx]

**Supplement B** Prediction of depressive symptoms at the 2.5 year follow-up (T1) by the interaction terms.

|  | Complete sample (T0) | | | |  | persons without elevated depressive symptoms at T0 | | |  | persons with elevated depressive symptoms at T0 | |
| --- | --- | --- | --- | --- | --- | --- | --- | --- | --- | --- | --- |
|  | PHQ-2^1^ ≥ 2 at T1 (2898/12484) | | | |  | PHQ-2 ≥ 2 at T1 (1455/9605) | | |  | PHQ-2 ≥ 2 at T1 (1443/2879) | |
|  | Adj. OR^2^ (95 % CI) | | p | |  | Adj. OR (95 % CI) | | p |  | Adj. OR (95 % CI) | p |
| **SES^3^** | |  | |  |  |  |  | |  |  |  |
| **Model 1** | |  | |  |  |  |  | |  |  |  |
| IA^4^ SES x sex | | 1.00 (0.98 – 1.02) | | 0.714 |  | 1.01 (0.98 – 1.03) | 0.700 | |  | 0.98 (0.95 – 1.02) | 0.983 |
| IA SES x partnership | | 1.02 (1.00 – 1.05) | | 0.075 |  | 1.04 (1.00 – 1.07) | 0.056 | |  | 1.00 (0.89 – 1.27) | 0.843 |
| IA SES x MMD^5^ | | 1.00 (0.97 – 1.02) | | 0.721 |  | 0.98 (0.95 – 1.01) | 0.134 | |  | 1.03 (0.99 – 1.07) | 0.153 |
| **Model 2** | |  | |  |  |  |  | |  |  |  |
| IA SES x sex | | 1.00 (0.98 – 1.02) | | 0.974 |  | 1.00 (0.98 – 1.03) | 0.837 | |  | 0.99 (0.95 – 1.03) | 0.565 |
| IA SES x partnership | | 1.02 (0.99 – 1.02) | | 0.247 |  | 1.03 (0.99 – 1.06) | 0.162 | |  | 1.01 (0.96 – 1.05) | 0.839 |
| IA SES x MMD | | 1.00 (0.97 – 1.02) | | 0.692 |  | 0.98 (0.95 – 1.01) | 0.162 | |  | 1.03 (0.98 – 1.07) | 0.229 |
| **Model 1** adjusted for sex, age, living with partner and major medical diseases.  **Model 2** adjusted for sex, age, living with partner, major medical diseases, PHQ-2 (T0), MH^6^ of depressive disorder, GAD-2 (T0) and MH of anxiety disorder.  ^1^PHQ-2 = interaction term  ^2^OR = Odds Ratio  ^3^SES = socioeconomic status  ^4^ IA = interaction term  ^5^ Major medical diseases = any of the following diseases: cardiovascular disease (CVD), cancer, diabetes, chronic obstructive pulmonary disease (COPD), asthma.  ^6^ MH = medical history. | | | | | | | | | | | |
|  | complete sample (T0) | | | |  | persons without elevated depressive symptoms at T0 | | |  | persons with elevated depressive symptoms at T0 | |
|  | (I) PHQ-2 ≥ 2 at T1 (2898/12484) | | | |  | (II) PHQ-2 ≥ 2 at T1 (1455/9605) | | |  | (III) PHQ-2 ≥ 2 at T1 (1443/2879) | |
|  | Adj. OR (95 % CI) | | p | |  | Adj. OR (95 % CI) | | p |  | Adj. OR (95 % CI) | p |
| **SES (low – medium – high)** | |  | |  |  |  |  | |  |  |  |
| **Model 1** | |  | |  |  |  |  | |  |  |  |
| IA^4^ SES three-part x sex | | 0.99 (0.87 – 1.14) | | 0.969 |  | 1.09 (0.91 – 1.31) | 0.363 | |  | 0.87 (0.69 – 1.11) | 0.263 |
| IA^4^ SES three-part x partnership | | 1.08 (0.92 – 1.27) | | 0.350 |  | 1.17 (0.93 – 1.46) | 0.176 | |  | 0.95 (0.72 – 1.24) | 0.698 |
| IA^4^ SES three-part x MMD | | 1.05 (0.90 – 1.23) | | 0.531 |  | 0.98 (0.79 – 1.20) | 0.824 | |  | 1.22 (0.93 – 1.60) | 0.144 |
| **Model 2** | |  | |  |  |  |  | |  |  |  |
| IA^4^ SES three-part x sex | | 1.01 (0.87 – 1.18) | | 0.863 |  | 1.07 (0.89 – 1.29) | 0.471 | |  | 0.90 (0.70 – 1.15) | 0.384 |
| IA^4^ SES three-part x partnership | | 1.03 (0.86 – 1.23) | | 0.753 |  | 1.12 (0.89 – 1.42) | 0.336 | |  | 0.95 (0.71 – 1.25) | 0.691 |
| IA^4^ SES three-part x MMD | | 1.07 (0.90 – 1.27) | | 0.423 |  | 1.01 (0.81 – 1.25) | 0.950 | |  | 1.19 (0.90 – 1.58) | 0.220 |
| **Model 1** adjusted for sex, age, living with partner and major medical diseases^3^.  **Model 2** adjusted for sex, age, living with partner, major medical diseases^3^, PHQ-2 (T0), MH^2^ of depressive disorder, GAD-2 (T0) and MH of anxiety disorder. | | | | | | | | | | | |

|  | complete sample (T0) | |  | persons without elevated depressive symptoms at T0 | |  | persons with elevated depressive symptoms at T0 | |
| --- | --- | --- | --- | --- | --- | --- | --- | --- |
|  | (I) PHQ-2 ≥ 2 at T1 (2898/12484) | |  | (II) PHQ-2 ≥ 2 at T1 (1455/9605) | |  | (III) PHQ-2 ≥ 2 at T1 (1443/2879) | |
|  | Adj. OR (95 % CI) | p |  | Adj. OR (95 % CI) | p |  | Adj. OR (95 % CI) | p |
| **Education** |  |  |  |  |  |  |  |  |
| **Model 1** |  |  |  |  |  |  |  |  |
| IA^4^ education x sex | 1.00 (0.96 – 1.04) | 0.824 |  | 1.02 (0.97 – 1.08) | 0.426 |  | 0-96 (0.89 – 1.03) | 0.253 |
| IA^4^ education x partnership | 1.01 (0.97 – 1.06) | 0.592 |  | 1.04 (0.97 – 1.11) | 0.276 |  | 0.99 (0.91 – 1.07) | 0.783 |
| IA^4^ education x MMD | 1.00 (0.95 – 1.05) | 0.953 |  | 0.96 (0.90 – 1.02) | 0.202 |  | 1.07 (0.98 – 1.17) | 0.122 |
| **Model 2** |  |  |  |  |  |  |  |  |
| IA^4^ education x sex | 1.00 (0.96 – 1.05) | 0.978 |  | 1.02 (0.97 – 1.08) | 0.487 |  | 0.97 (0.90 – 1.04) | 0.390 |
| IA^4^ education x partnership | 1.02 (0.96 – 1.07) | 0.574 |  | 1.03 (0.96 – 1.10) | 0.461 |  | 1.00 (0.92 – 1.09) | 0.988 |
| IA^4^ education x MMD | 0.99 (0.94 – 1.05) | 0.735 |  | 0.96 (0.89 – 1.03) | 0.218 |  | 1.06 (0.97 – 1.15) | 0.238 |
| **Model 1** adjusted for sex, age, living with partner and major medical diseases^3^.  **Model 2** adjusted for sex, age, living with partner, major medical diseases^3^, PHQ-2 (T0), MH^2^ of depressive disorder, GAD-2 (T0) and MH^2^ of anxiety disorder. | | | | | | | | |
|  |  | |  |  | |  |  | |
|  | complete sample (T0) | |  | persons without elevated depressive symptoms at T0 | |  | persons with elevated depressive symptoms at T0 | |
|  | (I) PHQ-2 ≥ 2 at T1 (2898/12484) | |  | (II) PHQ-2 ≥ 2 at T1 (1455/9605) | |  | (III) PHQ-2 ≥ 2 at T1 (1443/2879) | |
|  | Adj. OR (95 % CI) | p |  | Adj. OR (95 % CI) | p |  | Adj. OR (95 % CI) | p |
| **Occupational position** |  |  |  |  |  |  |  |  |
| **Model 1** |  |  |  |  |  |  |  |  |
| IA^4^ occupational position x sex | 0.97 (0.92 – 1.03) | 0.358 |  | 0.99 (0.92 – 1.06) | 0.708 |  | 0.94 (0.85 – 1.04) | 0.220 |
| IA^4^ occupational position x partnership | 1.06 (0.99 – 1.13) | 0.113 |  | **1.12 (1.01 – 1.23)** | **0.025** |  | 0.98 (0.87 – 1.10) | 0.679 |
| IA^4^ occupational position x MMD | 1.02 (0.96 – 1.08) | 0.584 |  | 0.97 (0.90 – 1.06) | 0.523 |  | 1.07 (0.96 – 1.19) | 0.203 |
| **Model 2** |  |  |  |  |  |  |  |  |
| IA^4^ occupational position x sex | 0.97 (0.92 – 1.04) | 0.385 |  | 0.97 (0.90 – 1.05) | 0.494 |  | 0.96 (0.87 – 1.06) | 0.405 |
| IA^4^ occupational position x partnership | 1.05 (0.97 – 1.13) | 0.216 |  | 1.09 (0.99 – 1.21) | 0.073 |  | 0.99 (0.88 – 1.12) | 0.848 |
| IA^4^ occupational position x MMD | 1.01 (0.95 – 1.08) | 0.724 |  | 0.98 (0.90 – 1.07) | 0.606 |  | 1.07 (0.96 – 1.20) | 0.238 |
| **Model 1** adjusted for sex, age, living with partner and major medical diseases^3^.  **Model 2** adjusted for sex, age, living with partner, major medical diseases^3^, PHQ-2 (T0), MH^2^ of depressive disorder, GAD-2 (T0) and MH^2^ of anxiety disorder. | | | | | | | | |
|  | complete sample (T0) | |  | persons without elevated depressive symptoms at T0 | |  | persons with elevated depressive symptoms at T0 | |
|  | (I) PHQ-2 ≥ 2 at T1 (2898/12484) | |  | (II) PHQ-2 ≥ 2 at T1 (1455/9605) | |  | (III) PHQ-2 ≥ 2 at T1 (1443/2879) | |
|  | Adj. OR (95 % CI) | p |  | Adj. OR (95 % CI) | p |  | Adj. OR (95 % CI) | p |
| **Income** |  |  |  |  |  |  |  |  |
| **Model 1** |  |  |  |  |  |  |  |  |
| IA^4^ income x sex | 1.02 (0.97 – 1.07) | 0.367 |  | 1.03 (0.96 – 1.10) | 0.392 |  | 1.01 (0.93 – 1.10) | 0.857 |
| IA^4^ income x partnership | **1.08 (1.01 – 1.16)** | **0.023** |  | 1.07 (0.97 – 1.18) | 0.165 |  | 1.07 (0.96 – 1.20) | 0.219 |
| IA^4^ income x MMD | **1.05 (1.03 – 1.07)** | **< 0.0001** |  | **1.05 (1.02 – 1.08)** | **0.002** |  | 1.02 (0.98 – 1.06) | 0.443 |
| **Model 2** |  |  |  |  |  |  |  |  |
| IA^4^ income x sex | 1.03 (0.98 – 1.09) | 0.297 |  | 0.89 (0.79 – 0.99) | 0.414 |  | 1.02 (0.93 – 1.11) | 0.705 |
| IA^4^ income x partnership | 1.04 (0.96 – 1.12) | 0.330 |  | 1.05 (0.95 – 1.16) | 0.348 |  | 1.04 (0.92 – 1.17) | 0.530 |
| IA^4^ income x MMD | 0.97 (0.92 – 1.04) | 0.412 |  | 0.94 (0.87 – 1.02) | 0.143 |  | 1.02 (0.92 – 1.13) | 0.673 |

**Model 1** adjusted for sex, age, living with partner and major medical diseases^3^.

**Model 2** adjusted for sex, age, living with partner, major medical diseases^3^, PHQ-2 (T0), MH^2^ of depressive disorder, GAD-2 (T0) and MH^2^ of anxiety disorder.
