# Supplementary material for: Income and education predict elevated depressive symptoms in the general population: results from the Gutenberg health study
Source: BMC Public Health. 2019 Apr 24;19:430. doi: 10.1186/s12889-019-6730-4 (PMC6480596; doi:10.1186/s12889-019-6730-4)
Supplement: Supplementary file 3 — Prediction of depressive symptoms at the 2.5 year follow-up (T1) by other variables. (DOCX 28 kb) [file 12889_2019_6730_MOESM3_ESM.docx]

**Supplement A** Prediction of depressive symptoms at the 2.5 year follow-up (T1) by other variables.

|  | Complete sample (T0) | | | |  | persons without elevated depressive symptoms at T0 | | |  | persons with elevated depressive symptoms at T0 | |
| --- | --- | --- | --- | --- | --- | --- | --- | --- | --- | --- | --- |
|  | PHQ-2^1^ ≥ 2 at T1 (2898/12484) | | | |  | PHQ-2 ≥ 2 at T1 (1455/9605) | | |  | PHQ-2 ≥ 2 at T1 (1443/2879) | |
|  | Adj. OR^2^ (95 % CI) | | p | |  | Adj. OR (95 % CI) | | p |  | Adj. OR (95 % CI) | p |
| **Model 1** | |  | |  |  |  |  | |  |  |  |
| Age (per year) | | **0.98 (0.97 – 0.98)** | | **< 0.0001** |  | **0.98 (0.97 – 0.98)** | **< 0.0001** | |  | **0.99 (0.98 – 0.99)** | **0.003** |
| Sex (m = 0, f = 1) | | **1.37 (1.25 – 1.50)** | | **< 0.0001** |  | **1.32 (1.18 – 1.48)** | **< 0.0001** | |  | **1.22 (1.05 – 1.42)** | **0.010** |
| Living with partner (1= yes) | | **0.76 (0.67 – 0.85)** | | **< 0.0001** |  | 0.88 (0.75 – 1.04) | 0.142 | |  | **0.70 (0.58 – 0.86)** | **0.001** |
| Major medical diseases^3^ (1 = yes) | | **1.30 (1.17 – 1.44)** | | **< 0.0001** |  | **1.34 (1.17 – 1.54)** | **< 0.0001** | |  | 1.06 (0.88 – 1.27) | 0.541 |
| Education (1-7) | | 0.99 (0.96 – 1.01) | | 0.269 |  | 0.98 (0.95 – 1.02) | 0.300 | |  | 0.98 (0.94 – 1.03) | 0.432 |
| Occupational position (range 1-7) | | **0.96 (0.93 – 0.99)** | | **0.018** |  | 0.96 (0.92 – 1.00) | 0.075 | |  | 1.01 (0.95 – 1.07) | 0.860 |
| Household net-income (range 1-7) | | **0.92 (0.89 – 0.95)** | | **< 0.0001** |  | **0.94 (0.90 – 0.98)** | **< 0.0001** | |  | 0.97 (0.92 – 1.03) | 0.351 |
| **Model 2** | |  | |  |  |  |  | |  |  |  |
| Age (per year) | | **0.98 (0.98 – 0.99)** | | **< 0.0001** |  | **0.98 (0.98 – 0.99)** | **< 0.0001** | |  | **0.99 (0.98 – 0.99)** | **0.032** |
| Sex (m = 0, f = 1) | | **1.16 (1.05 – 1.27)** | | **0.003** |  | **1.14 (1.01 – 1.29)** | **0.033** | |  | 1.10 (0.94 – 1.29) | 0.234 |
| Living with partner (1= yes) | | **0.87 (0.76 – 0.99)** | | **0.031** |  | 0.93 (0.78 – 1.10) | 0.401 | |  | **0.76 (0.62 – 0.94)** | **0.011** |
| Major medical diseases^3^ (1 = yes) | | **1.16 (1.03 – 1.30)** | | **0.015** |  | **1.26 (1.09 – 1.50)** | **0.002** | |  | 0.99 (0.82 – 1.19) | 0.901 |
| Education (1-7) | | **0.97 (0.94 – 0.99)** | | **0.015** |  | **0.96 (0.93 – 0.99)** | **0.036** | |  | 0.97 (0.93 – 1.02) | 0.206 |
| Occupational position (range 1-7) | | 0.97 (0.94 – 1.01) | | 0.162 |  | 0.97 (0.92 – 1.01) | 0.153 | |  | 1.00 (0.94 – 1.06) | 0.879 |
|  | | Complete sample (T0) | | |  | persons without elevated depressive symptoms at T0 | | |  | persons with elevated depressive symptoms at T0 | |
|  |  | PHQ-2 ≥ 2 at T1 (2898/12484) | | |  | PHQ-2 ≥ 2 at T1 (1455/9605) | | |  | PHQ-2 ≥ 2 at T1 (1443/2879) | |
|  |  | Adj. OR (95 % CI) | | p |  | Adj. OR (95 % CI) | p | |  | Adj. OR (95 % CI) | p |
| Household net-income (range 1-7) | | 0.97 (0.94 – 1.00) | | 0.062 |  | **0.96 (0.92 – 0.99)** | **0.046** | |  | 0.99 (0.94 – 1.05) | 0.782 |
| PHQ-2 at T0 (0 – 6) | | **1.78 (1.70 – 1.88)** | | **< 0.0001** |  | **2.03 (1.80 – 2.29)** | **< 0.0001** | |  | **1.22 (1.10 – 1.36)** | **< 0.0001** |
| MH^5^ of depressive disorder (1= yes) | | **1.65 (1.42 – 1.90)** | | **< 0.0001** |  | **1.95 (1.59 – 2.37)** | **< 0.0001** | |  | **1.40 (1.14 – 1.71)** | **0.001** |
| Anxiety at T0, GAD-2^4^ | | **1.40 (1.34 – 1.47)** | | **< 0.0001** |  | **1.48 (1.38 – 1.58)** | **< 0.0001** | |  | **1.31 (1.23 – 1.40)** | **< 0.0001** |
| MH of anxiety disorder (1= yes) | | **1.21 (1.01 – 1.44)** | | **0.038** |  | 1.06 (0.83 – 1.37) | 0.632 | |  | **1.36 (1.06 – 1.74)** | **0.015** |

**Coefficients from model** including education, occupational position and household net-income (range 1-7) + displayed variables. Please note that in the group of persons without depressive symptoms there is a range of 0 to 1 whereas in the group of persons with depressive symptoms there is a range of 2 to 6 due to separating both groups per PHQ-2 < vs. ≥ 2.

^1^PHQ-2 = Patient Health Questionnaire, depression module.

^2^OR = Odds Ratio..

^3^ Major medical diseases = any of the following diseases: cardiovascular disease (CVD), cancer, diabetes, chronic obstructive pulmonary disease (COPD), asthma.

^4^GAD-2 = Generalized Anxiety Disorder-Scale.

^5^MH = medical history.
